# Takotsubo syndrome‐associated ventricular standstill in a peripartum patient: case report and review of the literature

**DOI:** 10.1002/ccr3.1331

**Published:** 2017-12-22

**Authors:** Nelson Lee, Kevin Wade Lee, Matthew Michael D'Ambrosio, Joseph Vaughan Banta, Apostolos Voudouris, Antonios Tsompanidis

**Affiliations:** ^1^ Department of Cardiology/Family Medicine Residency Program CarePoint Health Christ Hospital Jersey City New Jersey; ^2^ Rowan University School of Osteopathic Medicine Stratford New Jersey

**Keywords:** Cardiomyopathy, pacemaker, peripartum, Takotsubo, ventricular standstill

## Abstract

Takotsubo syndrome is classically characterized by apical ballooning and left ventricle akinesis associated with an underlying catecholamine surge. In patients with suspected Takotsubo syndrome, clinicians should be vigilant for acute coronary syndrome and arrhythmias. Ventricular standstill with underlying Takotsubo syndrome should be managed with a dual‐chambered pacemaker to improve patient outcome.

## Introduction

Patients with Takotsubo syndrome (TTS) are well‐known to develop ventricular arrhythmias which predispose these patients to higher risks of life‐threatening complications 1. It is therefore important to highlight the development and medical management of these arrhythmias seen in patients with TTS. In cases with TTS, there have been few reported patients who have developed ventricular standstill (VS). Patients with ventricular standstill are prone to developing syncopal episodes due to the lack of ventricular depolarization caused by asystole of the cardiac conduction system leading to diminished cardiac output. It is suspected that ventricular ballooning from TTS impairs the cardiac conduction system such that the AV node and His‐Purkinje system can no longer effectively send impulses to the rest of the heart. Therefore, the vigilance of ventricular standstill and placement of a dual‐chambered pacemaker can help decrease the likelihood of unfavorable outcomes in patients with suspected TTS. We present a case of a peripartum patient who developed TTS and subsequent ventricular standstill.

## Case Report

A 24‐year‐old primiparous Egyptian female, 6 months status post vaginal delivery, presented to the emergency department with a two‐day history of chest pain and shortness of breath that worsened with exertion. Her past medical history included asthma and a history of one prior spontaneous abortion. She denied any history of smoking, alcohol, or drug use. She reported no known family history of cardiac disease, stroke, or malignancy.

**Figure 1:**
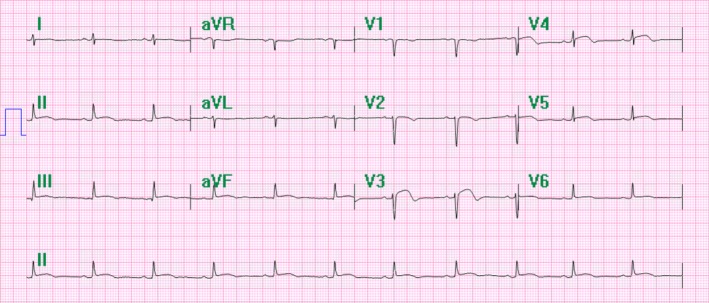
Initial EKG from first admission, ST elevations in V2–V4 and leads II, III, aVF. Write speed 10 mm/mV.

Upon initial presentation, the patient's vitals were within normal limits. Her temperature was measured at 98.2°F, heart rate of 86 beats per minute, blood pressure of 117/59 mmHg, and respiration rate of 14 breaths per minute. Initial electrocardiogram showed diffuse ST elevations in anterior leads (V2–V4) and inferior leads (II, III, aVF; Fig. 1). Subsequent electrocardiograms, performed at 1 and 2 h later, showed abnormalities similar to the first. Serial troponins were drawn every 8 h and found to be elevated at 2.69, 2.75, and 3.11 ng/mL (normal < 0.01 ng/mL), respectively. Initial portable chest X‐ray showed no active disease. Additionally, urine *β*‐hCG was negative and TSH was within normal limits.

**Figure 2:**
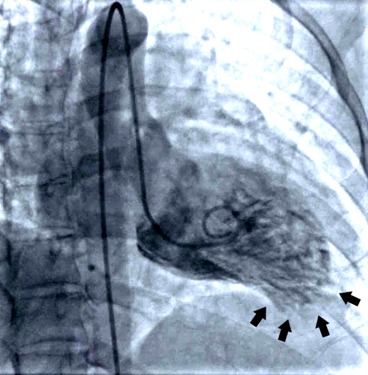
Akinesis and apical ballooning, both pathognomonic for Takotsubo syndrome, were seen during cardiac catheterization.

The patient was informed that she would need a cardiac catheterization; however, the patient refused due to concerns about maintaining her ability to breastfeed her infant child. She concluded that the benefits of breastfeeding her child outweighed the risk of contaminating her breast milk with the medications she was offered, so she refused them all except aspirin. The patient was then admitted to the intensive care unit for close observation. After further discussion, the patient finally agreed to undergoing cardiac catheterization which showed unremarkable coronary circulation with mild apical hypokinesis and apical ballooning with an ejection fraction of 55%, consistent with TTS, see Figure 2. D‐dimer was negative, as was the bilateral lower extremity dopplers. A 2‐D echocardiogram showed no regional wall motion abnormalities and an ejection fraction of 60%. Soon after being transferred out of the intensive care unit, the patient signed out of the hospital against medical advice, despite multiple discussions with the nursing staff and the interventional cardiologist, warning her of the risks of leaving.

**Figure 3:**
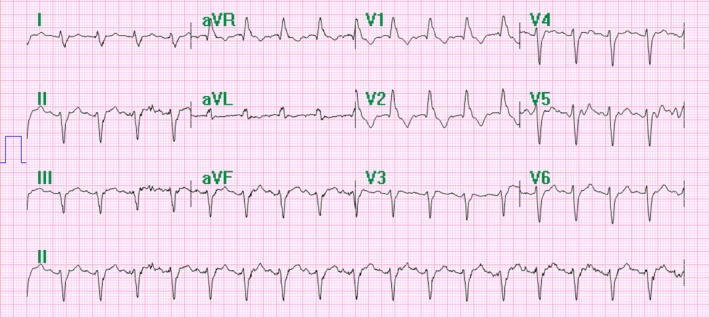
Initial EKG on second admission – sinus tachycardia with bifascicular block. Write speed 10 mm/mV.

**Figure 4:**
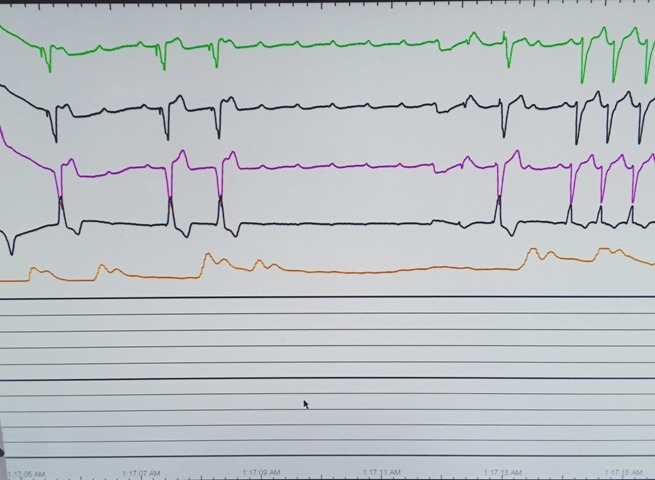
About 4 sec of ventricular standstill on telemetry while patient was coding in the intensive care unit.

**Figure 5:**
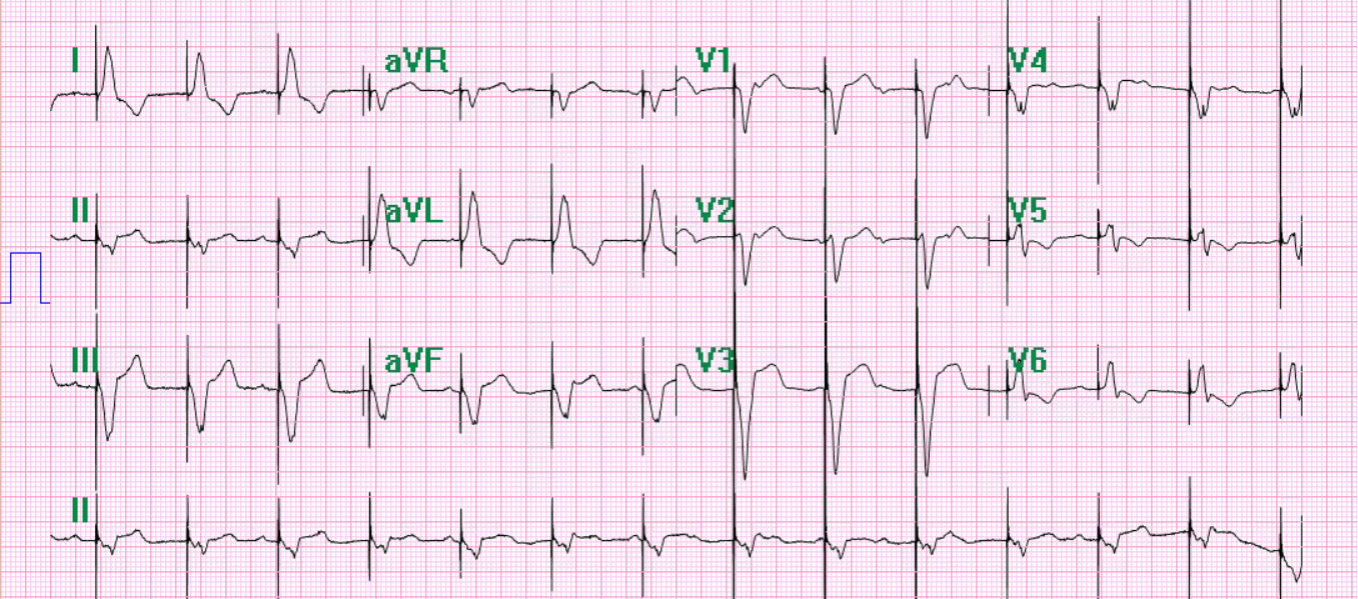
EKG after transvenous pacemaker placement showing appropriate ventricular pacing. Write speed 10 mm/mV.

The patient returned to the emergency room, 2 days later, complaining of recurrent syncopal episodes. The patient's ECG revealed sinus tachycardia with a bifascicular block (Fig. 3) and she was subsequently readmitted to the intensive care unit. The patient had a temporary transvenous pacemaker implanted, via transjugular approach, with atropine and epinephrine at bedside. Later that evening, the patient had three episodes of asystole and became unresponsive. She was given 1 mg of atropine and chest compressions for a few seconds before regaining consciousness. The patient was found to have ventricular standstill on telemetry at this time (Fig. 4). The patient's pacemaker was believed to not be capturing appropriately, and so the patient was taken to the catheterization laboratory for readjustment of her pacemaker under fluoroscopy, with the transjugular pacemaker being withdrawn and a new pacemaker placed via the transfemoral approach. Repeat ECG after pacemaker placement showed appropriate pacing, as shown in Figure 5. A troponin was collected and found to be elevated at 0.7590 ng/mL. Various laboratories including antinuclear antibody, rheumatoid factor, myeloperoxidase, proteinase 3, and rapid plasma reagin were all tested and found to be negative. Despite acute stabilization of the patient via pacemaker placement, an electrophysiology study was indicated given her recent syncope and the need to delineate the site of the heart block seen on telemetry. The patient was then transferred to a different hospital to undergo an electrophysiology study, which was negative for any cardiac conduction abnormalities. It was, however, determined that the patient would need a permanent pacemaker placed to prevent further syncopal episodes. A Sorin Reply DR dual chamber pacemaker (Serial no. 420ZU644) was inserted, with a Sorin R45 lead (Serial no. 29509097) placed in the atrium and Sorin R53 lead placed in the ventricle (Serial no. 29511696). Follow‐up echocardiograms at 1 and 8 months after discharge were normal with normal ejection fractions and the patient remained asymptomatic and stable. The patient's pacemaker interrogation from June 2016 shows she still requires atrial pacing 32% of the time. Her cardiologist reports similar numbers from more recent interrogations and states she remains asymptomatic at this time.

## Discussion

Takotsubo syndrome is a stress‐related cardiomyopathy in which weakening of the cardiac musculature leads to left ventricular failure with apical, midventricular, basal or focal akinesis, and ballooning 2. This is an atypical case of peripartum‐induced TTS that was further complicated by the presence of ventricular standstill, a rare complication of TTS. The patient in this case was believed to have experienced TTS secondary to her peripartum state. A low estrogen state is thought to be a predisposing factor for patients developing TTS 3. Literature has shown that patients suffering TTS were often identified as being postmenopausal women. Because this patient opted to breastfeed, we believe that her physiologically low estrogen state may have been a significant predisposing factor to developing TTS. A case presented by Sato et al. 4 discussed a 41‐year‐old patient that developed TTS after delivery, attributed to that fact that she had Turner's syndrome, which makes her estrogen levels considerably lower than normal females. During the workup for this patient, multiple autoimmune and infectious etiologies were ruled out by testing antinuclear antibody, rheumatoid factor, myeloperoxidase, proteinase 3, and rapid plasma reagin. While there was a high suspicion for Takotsubo syndrome in this patient during initial workup, we believed it necessary to rule out additional infectious and autoimmune causes of cardiomyopathy.

While the underlying etiology of ventricular standstill (VS) has been attributed to an increase in vagal tone, few studies have shown Takotsubo syndrome as an underlying mechanism for VS. It is suspected that complete conduction failure through the AV node and His‐Purkinje system resulted as a consequence of apical ballooning and regional wall abnormalities, leading to the observed bifascicular block, which progressed to VS. As a result, the automaticity of the ventricle was inhibited, manifesting as recurrent syncopal episodes. It is also possible that rapid and elevated concentrations of catecholamines such as norepinephrine can cause intense vasoconstriction and multiple‐branch coronary artery spasms 2. Ultimately, persistent spasms of multiple‐branch coronary arteries may lead to continuous ischemic damage and eventual conduction pathway fibrosis leading to rapid deterioration of the cardiac conduction system and an impaired ability to communicate electrical impulses through the circuit. In our patient, serum troponins were elevated suggesting underlying ischemic damage 6.

Literature review revealed only one other documented case of TTS with ventricular standstill in a 66‐year‐old patient who was believed to have had TTS secondary to epidural placement prior to surgery for an adrenal mass 6. The pathophysiology of bradyarrhythmias in TTS is still unclear, although several proposed mechanisms include reduced coronary blood flow to conduction pathways secondary to ventricular dyskinesia, catecholamine‐induced coronary vasospasm, and conduction pathway fibrosis secondary to prolonged ischemia 6. Due to the presence of ventricular standstill and bifascicular block in our patient, a permanent pacemaker was deemed necessary to minimize the risk of death caused by fatal arrhythmias. In a retrospective study conducted between 2004 and 2013 by Stiermaier et al. patients who underwent permanent pacemaker implantation were found to have persistent high‐degree AV block, despite complete recovery of ventricular function 2. Numerous published studies support implantation of pacemaker in the presence of persistent AV block 2, 7, 8. Despite this, there are no definite indications in the current guidelines to suggest a mortality benefit with permanent pacemaker placement in ventricular standstill. Current guidelines, however, recommend permanent pacemaker placement for significant clinical benefit in diseases associated with the His‐Purkinje system as these patients are considered to be less stable and more likely to progressively worsen 9.

Due to the typically transient nature of TTS, the prognosis of patients tends to be favorable. However, a recent prospective study reported that the prevalence of life‐threatening arrhythmias is higher than previously reported 10. These reported arrhythmias consist of ventricular tachycardia, ventricular fibrillation, asystole, and complete AV block. Management of these arrhythmias varied on a case by case basis based on patient characteristics, type, and context of the arrhythmic event 10. This patient showed progressive electrical instability with the onset of a new right bundle branch block and left anterior fascicular block with eventual standstill of conduction in a peripartum patient. This case report represents the first example of suspected septal pathology as an underlying mechanism in TTS‐associated VS, which will warrant further investigation in future studies. Management with pacemaker placement in those suspected with life‐threatening arrhythmias in TTS is essential as this can contribute to improved outcome in patients.

## Conclusion

This is the first case report of Takotsubo syndrome found in a peripartum patient with subsequent ventricular standstill. Permanent pacemaker placement should be considered for patients that suffer from such severe conduction abnormalities.

## Authorship

NL, KWL, MMD, JVB: contributed to drafting and editing of the manuscript as well as contributing the images/videos; AV: contributed to the evaluation and management of the patient, advised on technical details of the case, as well as made expert critiques on details of the manuscript. AT: contributed to the editing and expert critiquing of the manuscript.

## Conflict of Interest

None declared.

## Supporting information


**Video S1.** Visualization of LAD and circumflex.

**Video S2.** Visualization of RCA.

**Video S3.** Left ventricular angiogram.
